# Palindromic amplification of the *ERBB2* oncogene in primary HER2-positive breast tumors

**DOI:** 10.1038/srep41921

**Published:** 2017-02-17

**Authors:** Michael Marotta, Taku Onodera, Jeffrey Johnson, G. Thomas Budd, Takaaki Watanabe, Xiaojiang Cui, Armando E. Giuliano, Atsushi Niida, Hisashi Tanaka

**Affiliations:** 1 Lerner Research Institute and Cleveland Clinic, Cleveland, OH, USA; 2 Institute of Medical Science, University of Tokyo, Tokyo, Japan; 3 Department of Surgery, Cedars-Sinai Medical Center, West Hollywood, CA, USA; 4 Taussig Cancer Institute, Cleveland Clinic, Cleveland, OH, USA

## Abstract

Oncogene amplification confers a growth advantage to tumor cells for clonal expansion. There are several, recurrently amplified oncogenes throughout the human genome. However, it remains unclear whether this recurrent amplification is solely a manifestation of increased fitness resulting from random amplification mechanisms, or if a genomic locus-specific amplification mechanism plays a role. Here we show that the *ERBB2* oncogene at 17q12 is susceptible to palindromic gene amplification, a mechanism characterized by the inverted (palindromic) duplication of genomic segments, in HER2-positive breast tumors. We applied two genomic approaches to investigate amplification mechanisms: sequencing of DNA libraries enriched with tumor-derived palindromic DNA (Genome-wide Analysis of Palindrome Formation) and whole genome sequencing (WGS). We observed significant enrichment of palindromic DNA within amplified *ERBB2* genomic segments. Palindromic DNA was particularly enriched at amplification peaks and at boundaries between amplified and normal copy-number regions. Thus, palindromic gene amplification shaped the amplified *ERBB2* locus. The enrichment of palindromic DNA throughout the amplified segments leads us to propose that the *ERBB2* locus is amplified through the mechanism that repeatedly generates palindromic DNA, such as Breakage-Fusion-Bridge cycles. The genomic architecture surrounding *ERBB2* in the normal genome, such as segmental duplications, could promote the locus-specific mechanism.

Genome instability is an enabling characteristic by which tumor cells acquire unlimited proliferation and metastatic potential^1^. Instability can occur either in a small number of nucleotides (mutations) or in the organization of large genomic segments (gross chromosomal rearrangements, GCR). Among GCRs, an abnormal accumulation of genomic segments harboring oncogene (oncogene amplification) is associated with advanced stage disease and confers therapy resistance^2^,^3^,^4^,^5^. There are several recurrent oncogene amplifications throughout the human genome^6^. Cytogenetically, genomic segments can either accumulate extra-chromosomally in the form of mini-chromosomes (double minute chromosomes) or can cluster locally within chromosomes (intra-chromosomal homogenously staining regions)^7^. A number of models for gene amplification mechanisms have been proposed based on results from experimental model systems, such as *in vivo* mouse models, mammalian cell systems and more robust genetic systems of simple organisms^8^,^9^,^10^,^11^,^12^,^13^,^14^,^15^,^16^. However, whether these mechanisms underlie clinically-relevant, recurrent gene amplification in primary tumors remains elusive.

A well-recognized mechanism of gene amplification is Breakage-Fusion-Bridge (BFB) cycles^8^,^10^,^12^,^17^,^18^,^19^,^20^, originally described as a fate of chromosomes with two centromeres (dicentric chromosomes) by Barbara McClintock in 1941^21^. Dicentric chromosomes can arise from either (1) telomere-telomere fusions between chromosomes with critically short telomeres (hetero-dicentric) or (2) fusions of two broken sister chromatids at the broken ends (iso-dicentric) (Fig. 1a). During mitosis, each centromere moves to opposite poles, resulting in a break (at a random location on the chromosome arm). A broken chromosome could continue BFB cycles by forming an iso-dicentric chromosome after replication^10^,^18^. Because genomic segments would be unevenly inherited by daughter cells due to the random locations of breaks, repeating this cycle would lead to a population of cells with heterogeneous copy numbers (copy number heterogeneity) (Fig. 1b and c). Accordingly, genomic segments amplified by BFB cycles would exhibit two genomic signatures: palindromic, fold-back inversions at fusion points and copy number heterogeneity. Recurrent oncogene amplification that satisfies these two signatures is a candidate for BFB cycle-driven amplification.

Copy number heterogeneity has repeatedly been reported for the amplification of the epidermal growth factor receptor (HER2) gene *ERBB2* at 17q12–21.1 in breast tumors^22^,^23^,^24^. Breast tumors with *ERBB2* amplification constitute an aggressive, HER2-positive subtype that accounts for 15–20% of breast tumors^3^,^25^. The amplification of *ERBB2* causes the overexpression of HER2 that promotes cell proliferation signaling. Intensive efforts have been made to improve the outcome of this subtype, and we now have targeted diagnostic tests and therapies. Immunohistochemical staining of biopsy and surgical specimens for HER2 protein is a routine screening test for the HER2-positive subtype, with confirmation by fluorescence *in situ* hybridization (FISH) for increased copy numbers of *ERBB2* relative to the chromosome 17 centromere^26^,^27^. Amplified HER2 is targeted with FDA-approved monoclonal antibodies such as trastuzumab and pertuzumab that significantly improve patient outcomes^28^,^29^,^30^,^31^. Despite such success in clinical applications, little progress has been made in describing the mechanism causing *ERBB2* amplification. Mechanistic insights may help us to better understand the cancer etiology and to provide a novel insight underlying the current problems associated with targeted monoclonal antibody therapy, including both the *de novo* and acquired resistance^32^,^33^,^34^,^35^.

In this study, we seek to determine the mechanism of *ERBB2* amplification in primary breast tumors. Because *ERBB2* copy numbers are extensively heterogeneous between individual tumor cells (Fig. 2), BFB cycles are a strong candidate for the underlying mechanism. Thus, our approaches were intended to determine another signature of BFB cycles: palindromic fold-back inversions at fusion points. We found that fold-back inversions were distributed throughout the amplified regions, indicating that (1) inversions occur many times during the establishment of amplified genomic segments, and (2) fusion points may differ between individual tumor cells (breakpoint heterogeneity). These results strongly suggest that BFB cycles underlie the amplification of *ERBB2*.

## Results

### Strategy to identify palindromic fold-back inversions

**Figure 2:**
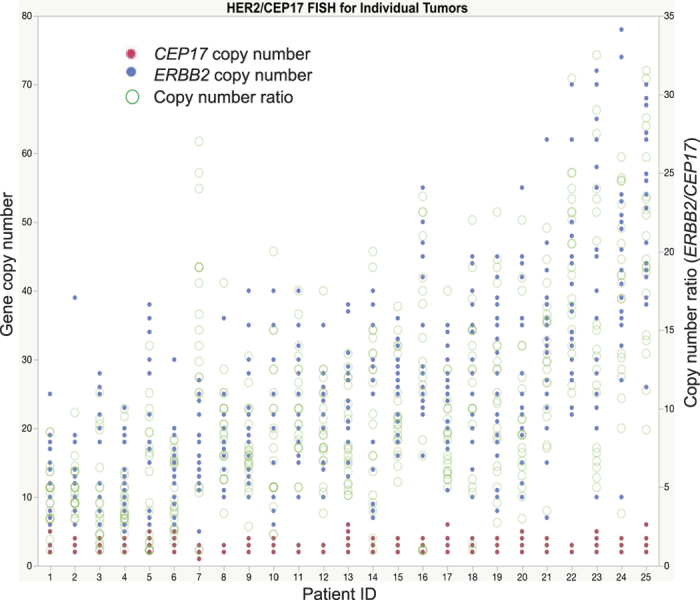
Extensive copy number heterogeneity of the *ERBB2* gene in HER2-positive breast tumors. Gene copy number (y-axis) was determined by counting FISH signals in 20 cells from each tumor (x-axis). Red dots indicate the number of signals of chromosome 17 centromere (CEP17) per cell and blue dots indicate the number of *ERBB2* signals per cell. Green circles represent the copy number ratio (ERBB2/CEP17) in each cell. Dot size corresponds to the number of cells.

Previous studies reported that a common pattern of copy number transitions of chromosome 17 is associated with *ERBB2* amplification in HER2 positive breast tumors: 17p loss, 17q12-21.1 amplification with the loss of flanking regions, and distal 17q gain^36^,^37^. We confirmed the common pattern in the copy-number profiles from the 117 HER2-positive breast tumors obtained from The Cancer Genome Atlas^25^ (Supplementary Fig. 1). Chromosome-wide copy-number alterations could occur in two ways. Continuously-occurring chromosome breaks and rearrangements during tumor cell proliferation could establish complex alterations step by step. Alternatively, rearrangements could occur all at once and establish complex alterations, as previously proposed for complex rearrangements triggered by chromothripsis^38^. However, because *ERBB2* copy number is heterogenous in a tumor cell population (Fig. 2), independent rearrangements occur repeatedly in each cell. It is unlikely that a single event followed by a clonal expansion could produce such heterogeneous cell populations. Hence, we considered a mechanism that (1) establishes gene amplification step by step and (2) creates copy number heterogeneity.

In this regard, breakage-fusion-bridge (BFB) cycles are a candidate mechanism, which causes copy number heterogeneity^21^. To test this possibility, we investigated another signature of BFB cycles, palindromic, fold-back inversions within amplified *ERBB2* genomic region^39^. We first employed our established method to enrich palindromic DNA from genomic DNA (Supplementary-Fig. 2)^11^,^39^,^40^,^41^,^42^. We denatured genomic DNA by boiling and quickly renatured it by cooling on ice. We have shown previously that, in the presence of 100 mM NaCl in the reaction, palindromic DNA efficiently folds back intra-molecularly and forms double-stranded DNA whereas non-palindromic DNA remains single-stranded^42^. Treatment with single-stranded nucleic acids-specific nuclease S1 eliminates single-stranded DNA and, as a result, enriches double-stranded DNA from palindromic, fold-back inversions. By sequencing the libraries derived from enriched DNA, we can identify genomic locations of palindromic DNA (Genome-wide Analysis of Palindrome Formation, GAPF-seq). Libraries made from enriched DNA have reduced complexity and thus allow us to identify the locations of somatic palindromic breakpoints without sequencing the libraries very deeply.

### Palindromic, fold-back inversions for the amplification of *ERBB2*

**Figure 3:**
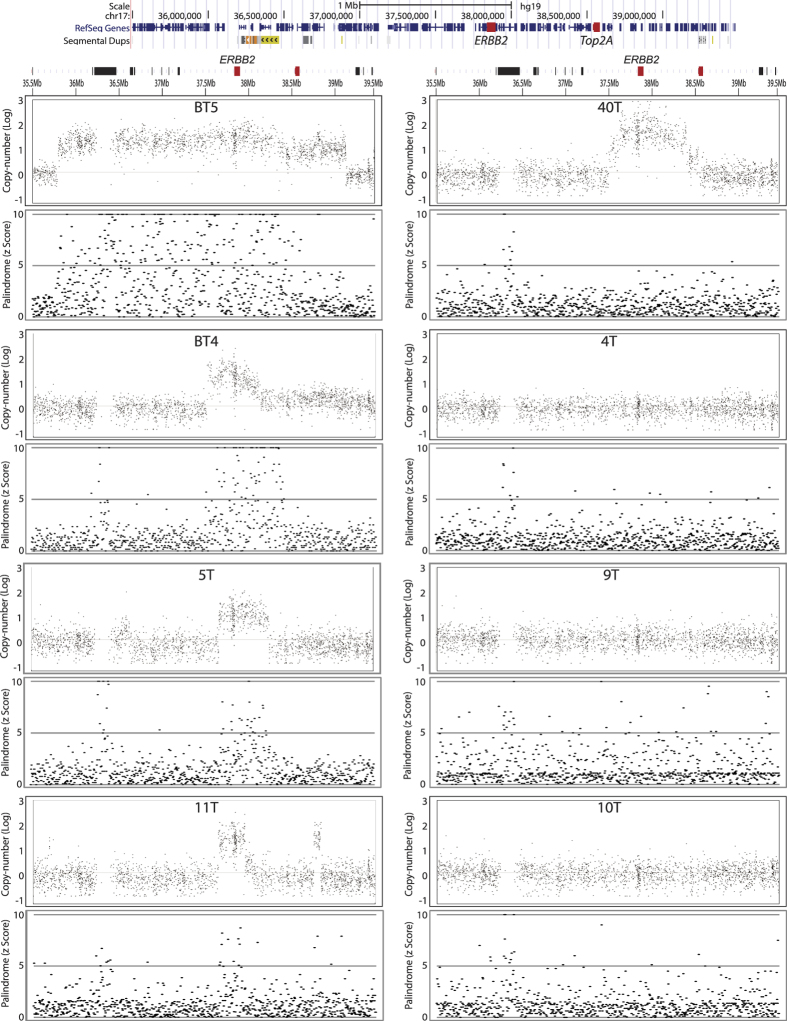
Copy number alterations (top panels) and the enrichments of palindromic DNA (bottom panels) in the 4 Mb *ERBB2* gene locus in DNA from 8 breast tumors. The 4 Mb region harboring *ERBB2* gene is shown on the top. For each tumor, log-ratio copy number relative to normal DNA is shown on the top and read depth of palindromic DNA is shown on the bottom. Read depth of palindromic DNA is shown using z-score (departure from the mean depth of the entire genome) in each 5 kb bin.

Genomic DNA from 8 breast tumor samples was included in this study. The status of HER2 was clinically determined by diagnostic FISH: five tumors were HER2-positive tumors (BT4, BT5, 5 T, 11 T and 40 T) and three were HER2-negative tumors (4 T, 9 T, and 10 T). Genomic DNA was processed (1) for conventional array-CGH using high-density oligonucleotide microarray (Affymetrix SNP 6.0) and (2) for GAPF-seq (Fig. 3). The copy number profiles confirmed clinical diagnosis; all HER2-positive breast tumors had at least four fold amplification of 17q12-21, whereas copy numbers were normal for all HER2-negative tumors. The sizes of amplified segments varied between samples, from 0.4 Megabase (Mb) (11 T) to 3.4 Mb (BT5). Also, amplified segments were very complex in two tumors (BT5 and 11 T). There were two copy number peaks in these tumors that flank the region of normal copy number (11 T) or the region of low-level amplification (BT5).

For the enrichment of palindromic DNA, we measured the read depth (the number of sequencing reads) in every 5-kb window (bin) of the entire genome. For each bin, we calculated a z-score. The z-score represents the number of standard deviations from the mean of the read depth, with a positive z-score indicating enrichments of reads in a given 5-kb bin. Bins with high z-score (>5) were very common and were distributed throughout the amplified genomic segments in 4 HER2-positive tumors (BT4, BT5 5 T, and 11 T). However, within an amplified genomic segment, only a subset of bins showed high z-score (>5) and many of them were close to the mean. This was in sharp contrast to array-CGH in which a vast majority of signal measurements (probes) within an amplified segment displayed high intensity (copy number gains). This difference confirmed that two approaches measured different genomic features: copy numbers and structures of DNA^39^,^42^. Palindromic DNA only existed where DNA rearrangements occurred within amplified segments.

We also noticed that some genomic segments exhibited only one of the two features. There was a segment with high-copy-number without palindromic DNA (bins with z-scores >5, 38.5–39 Mb region in BT5), and a region harboring palindromic DNA that was not amplified (38.2–38.5 Mb in BT4). Finally, in 40 T, palindromic DNA was very rare throughout the amplified segments (Fig. 3, top right). Thus, palindromic DNA was associated with a majority but not all *ERBB2* amplification. The region lacking copy number probes (SNP markers, 36.3–36.4 Mb) had a cluster of bins with high z-scores. This region is enriched with segmental duplications, recent large duplications in the human genome that have very high sequence identities (>90%) between duplications (Supplementary Fig. 3). Enrichment of palindromic DNA in all eight samples may indicate preexisting, germline palindromic DNA (inverted duplications) within the region.

Taken together, there was a strong co-occurrence between copy number increase and palindromic DNA within the *ERBB2* region in HER2-positive breast tumors.

### Palindromic DNA and the loss of chromosome 17p

**Figure 4:**
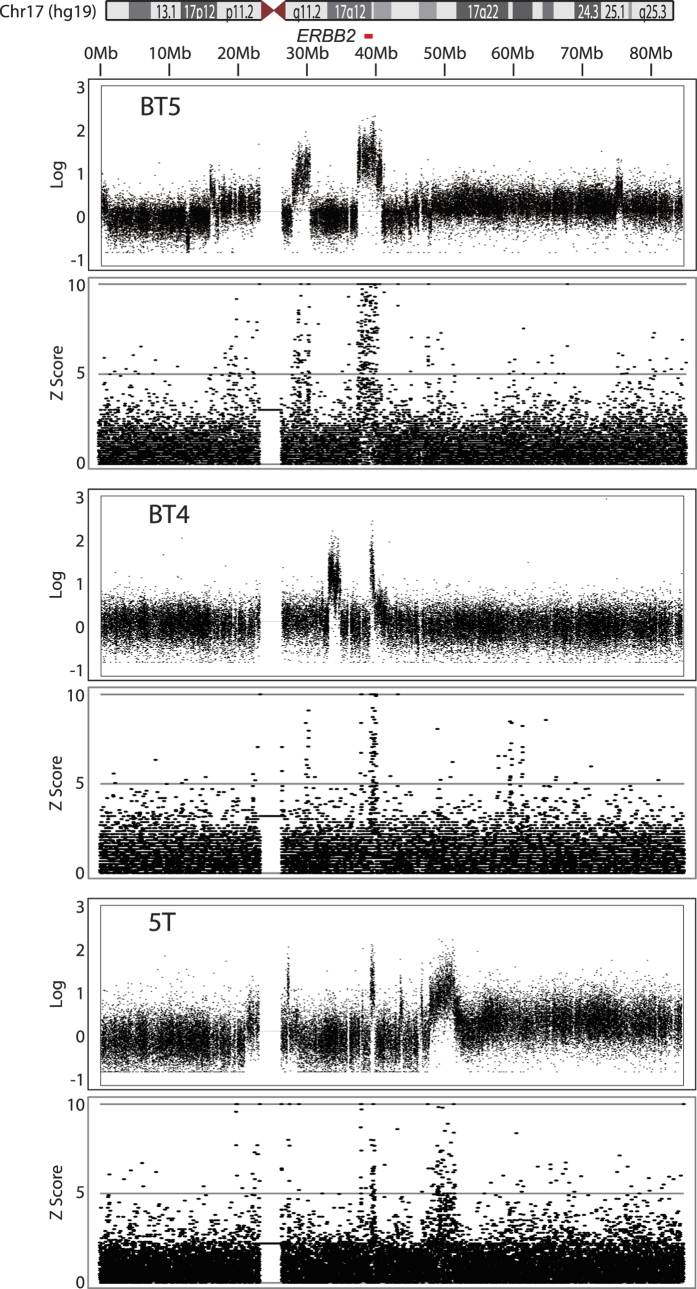
Copy number alterations and the enrichments of palindromic DNA for the entire chromosome 17. log ratio copy number relative to normal DNA is shown on the top and the read depth of palindromic DNA (z-score) is shown on the bottom.

We next addressed whether the co-occurrence between palindromic DNA and copy number alterations was limited to the *ERBB2* genomic segments or was widespread throughout chromosome 17 (Fig. 4). All the HER2-positive tumors had additional focal copy number alterations within chromosome 17. High-level copy number gains were restricted to the long arm in all cases (Supplementary Fig. 4). We noticed that many but not all the focal amplifications were associated with palindromic DNA (bins with high z-score). For example, palindromic DNA was enriched in the large amplified region near the centromere in BT5 and at the 45–50 Mb region in 5 T. In contrast, the focal amplification of the 35 Mb region in BT4 was not associated with palindromic DNA. In BT4, there were three peaks of palindromic DNA both at the 30 Mb and between the 50–60 Mb regions that were not accompanied by copy number gains.

The loss of short arm (17p) was common in HER2-positive breast tumors (Supplementary Fig. 1). By a closer look, we noticed that the formation of palindromic DNA might also be associated with 17p loss. We did not observe tumors with complete short arm loss (Supplementary Fig. 4). In four tumors with palindromic *ERBB2* amplification (5 T, BT4, BT5, and 11 T), most parts of chromosome 17 were lost; however, some segments were either retained or amplified at a low level. In BT5 and 5 T, the centromere-proximal region of 17p showed low-level copy number gains (Fig. 4). In both tumors, palindromic DNA was enriched in those regions. In BT4, a very small segment adjacent to the centromere gained copy number and exhibited the enrichment of palindromic DNA. Finally, the 17p loss was not seen in 40 T in which *ERBB2* amplification was not associated with palindromic DNA (Supplementary Fig. 4).

These data imply that there were, at least, two, patterns of copy number gains and losses of chromosome 17 in HER2-positive breast tumors. A dominant pattern (in 5 T, BT4, BT5, and 11 T) indicate the co-occurrence of palindromic DNA and copy number gains/losses throughout chromosome 17. In a unique pattern (40 T), copy number gains were independent of palindromic DNA. BFB cycles and the formation of palindromic DNA may play a role in the loss of 17p (and thus the loss of tumor suppressor *TP53* located in 17p).

### Palindromic DNA at an amplification peak and amplicon boundaries

**Figure 5:**
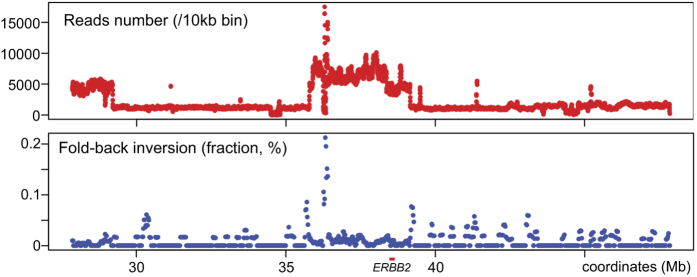
Copy numbers (top) and fold-back inversions (palindromic DNA) obtained from the whole genome sequencing data of BT5 tumor DNA. Results for the 20 Mb region flanking *ERBB2* is shown. Copy number alterations are shown by the read depth and fold-back inversions are shown as a fraction of total reads for each 10 kb bin.

We independently tested the enrichment of palindromic DNA within the amplified *ERBB2* genomic segments by investigating fold-back inversions in paired end-reads from conventional whole genome sequencing (WGS). We chose the breast tumor BT5 for WGS because BT5 had large amplified regions that were enriched with palindromic DNA (Fig. 5). Genomic DNA fragments of approximately 250 bp were processed to construct a library. Approximately 452 million fragments were sequenced from both ends (50 bp paired-end sequencing). In cases of normal paired-end reads, two ends are mapped onto opposite strands, and the distance between the two mapped ends equals the size of genomic DNA fragments processed for the library construction. In contrast, in palindromic DNA, two ends are mapped onto the same strands, and the distance between two ends are variable, depending on the distance between rearranged segments (fold-back inversions). We looked for the paired-ends that were aligned on the same strand and were less than 10-kb apart. We determined the enrichment of palindromic DNA (red) for each 10 kb bin as the fraction of paired ends mapped to the same strand divided by the number of total paired ends (blue).

Read depth analysis for the 20 Mb region surrounding *ERBB2* (Chr 17: 27.844,393–47,851,434) revealed a far more complex pattern of copy number alterations than array-CGH analysis (Fig. 5). There was a sharp copy-number peak on the centromeric side within the amplified segments flanked by a small normal copy-number segment on both sides. This copy-number peak was associated with the cluster of 10 kb bins enriched with fold-back inversions. Thus, this copy number peak was likely driven by the formation of palindromic DNA. We also noticed that both the centromeric and telomeric boundaries of the whole amplicon were marked by the peaks of fold-back inversions. These results were consistent with our previous observation by GAPF, in which palindromic DNA sets the boundaries of an amplicon^11^,^39^. Finally, although fold-back inversions were detected throughout the amplified segments, the frequency was very low for each bin. An explanation for this low-level enrichment was that fold-back inversions were not clonal, and their locations were heterogeneous within a tumor cell population. This heterogeneity would further support the BFB cycles for an underlying mechanism of *ERBB2* amplification.

Recently, six rearrangement signatures across breast tumors have been identified based on the WGS data^43^. Whether a particular rearrangements pattern underlies *ERBB2* amplification remains unclear, given the signatures are based on the rearrangements detected in the entire genome. To address this issue, we examined the WGS dataset (European Genome-Phenome Archive, EGAD00001001334). We first calculated HER2 copy number ratio (CN ratio) between tumor and matched normal samples and called HER2-positive when log2 (CN_tumor/CN_normal) was more than 2. For the tumors that satisfied the criteria (30 tumors/normal pairs), we looked for fold-back inversions using Genomon-SV platform^44^. Briefly, Genomon-SV uses information from both chimeric reads and discordant paired-end reads to call candidate variants. High confidence somatic variants are called after removing (i) variants in normal samples, (ii) variants only covered by a few reads and (iii) variants with low allele frequency. Thus, the structural variants called by Genomon-SV exist only in the tumors with multiple reads spanning the breakpoints.

**Figure 6:**
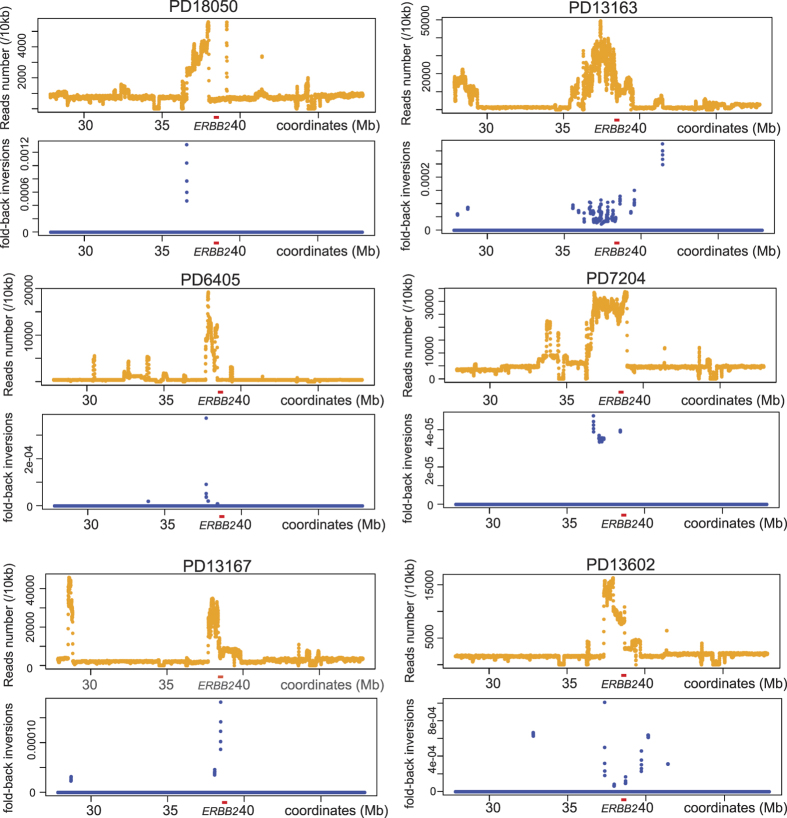
Fold-back inversions in the *ERBB2* locus in the publically-available WGS data sets of HER2-positive breast tumors^43^. Focal copy number increases (top) and the fraction of fold-back inversions (bottom) are presented for the 20 Mb region surrounding *ERBB2*.

We determined the fractions of reads representing fold-back inversions in each 10 kb bin in the 20 Mb region surrounding *ERBB2* (Fig. 6), as we did for the Fig. 5. We found that fold-back inversions were co-localized with amplified *ERBB2* segments in 13 tumors. Fold-back inversions were only detected in a limited number of bins, which indicates that only a small number of fold-back inversions were represented by multiple reads covering breakpoints. We also found seven tumors in which fold-back inversions existed within the 20 Mb locus but not co-localized with the *ERBB2* amplicon. Ten tumors did not have fold-back inversions within the 20 Mb. These results indicate the association between fold-back inversions and *ERBB2* genomic locus.

## Discussion

Although the role of oncogene amplification in tumorigenesis is well-established and separately, the molecular mechanisms underlying palindromic gene amplification have been extensively studied^8^,^10^,^11^,^12^,^14^,^45^, a strong link has not previously been made between a molecular amplification mechanism and the etiology of a particular cancer type. Our results show that palindromic gene amplification, likely resulting from BFB cycles, is a predominant mechanism driving *ERBB2* oncogene amplification in HER2-positive breast tumors. It appears that the incidence of HER2-related tumors is driven not only by the relative fitness advantage conferred by the overexpression of HER2 and other genes within the amplicon^36^ but is also a result of a specific amplification mechanism. This is in line with other mounting evidence suggesting that rather than being random, many of the genetic abnormalities resulting in cancer may in part be due to intrinsic features of the genomic, genetic and epigenetic substrate as well as protein machinery that interacts with it^46^,^47^.

What makes the *ERBB2* locus prone to palindromic gene amplification? There are a number of local genomic features in this locus that would promote palindromic gene amplification. The *ERBB2* locus is surrounded by large blocks of repeated (duplicated) DNA where long inverted repeats (>1 kb) are abundant (Supplementary Fig. 3)^48^. We and others have shown that inverted repeats promote palindromic gene amplification^8^,^10^,^11^,^49^. Repeat length is a factor for intra-strand annealing and longer repeats are more prone to intra-strand annealing. There is a large block (500 kb) of duplicated DNA clustered on the 1.2 Mb centromeric side of *ERBB2*. On the telomeric side, there is another cluster consisting of keratin (*KRT*) and keratin-associated protein (*KRTAP*) gene families. We have previously mapped a common copy number breakpoint of the *ERBB2* amplicon within this gene cluster, suggesting a functional role of duplicated DNAs in *ERBB2* amplification^48^. Moreover, there are two regions exhibiting discordance between genome assemblies and lacking single-nucleotide polymorphism (SNP) markers. One of the regions is located on the centromeric side (17q12) of *ERBB2* and spans for 2 Mb. Another one on the telomeric side covers a 100 kb region at 17q21.31. Both regions also harbor segmental duplications. Such local genomic complexity again indicates the abundance of inverted repeats. Within those complex regions, inverted repeats can disturb replication fork movements (Supplementary Fig. 5)^50^. Unresolved replication stress can lead to fork collapse and DSBs.

**Figure 7:**
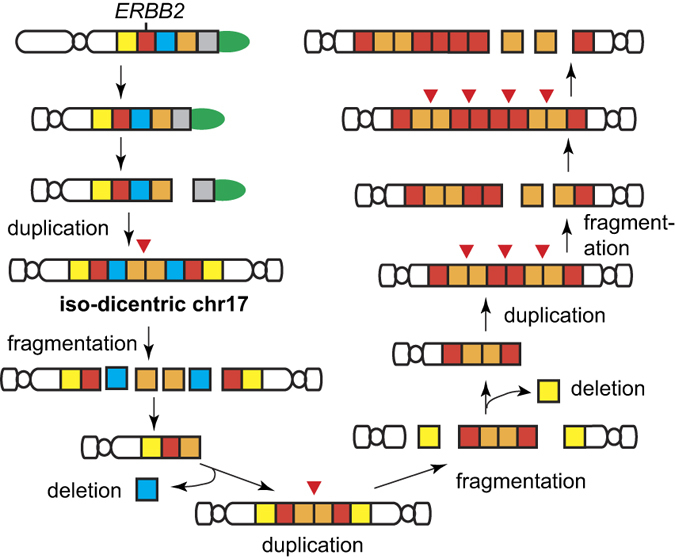
Amplification mechanism of *ERBB2* (model). A chromosome-level mechanism of duplication-fragmentation-deletion cycles for the amplification of *ERBB2*.

With these mechanistic insights, we propose a model in which BFB cycles give rise to *ERBB2* amplification in HER2-positive breast tumors (Fig. 7). *TP53* mutations and loss of 17p may precede the BFB cycles because the loss of TP53 function allows cells to proliferate with structurally abnormal DNA and to undergo gene amplification^51^,^52^. An initiating event can occur at the preexisting DNA inverted repeats on 17q. A resulting hairpin-capped chromosome would become a dicentric chromosome after replication (Supplementary Fig. 5). When each centromere is pulled to different daughter cells, the chromosome would become fragmented (see the discussion below) and cause deletions of the segment next to *ERBB2* segment. This rearranged chromosome with a broken end would again become a dicentric chromosome after replication. Repeating this duplication-fragmentation- deletion cycle would produce a progenitor cell in which one chromosome harbors multiple *ERBB2* genomic segments.

Very recent basic research has begun to reveal molecular mechanisms of BFB cycles in detail. It has been argued that a simple mechanical force by segregating centromeres into opposite pole is not sufficient to break a dicentric chromosome^53^. Rather, dicentric breakage was shown to require cytokinesis in yeast^54^. Either pharmacological or genetic suppression of a contractile ring at cell division prevents dicentric breakage. Another mechanistic insight on dicentric breakage came from human cells in which dicentric chromosomes formed by the fusion of critically short telomeres^55^. In this system, dicentric breakage depends on the cytoplasmic 3′ nuclease TREX1. Dicentric chromosomes developed into chromatin bridges after mitotic exit and underwent nuclear envelope rupture during interphase when TREX1 localized in chromatin bridges to create single-stranded DNA-binding protein (RPA)-coated ssDNA. Because TREX1 localize throughout the bridges, multiple breakage and fragmentation is possible (Fig. 7). Basic research has also made a significant progress for defining a molecular mechanism of chromosome fusion. Intra-strand annealing between inverted repeats now has a support from strong genetic and molecular studies in yeast in which failure to protecting the 3′ tail after end resection of DSBs by ssDNA binding protein RPA causes intra-strand annealing and the fold-back hairpin structure^14^,^56^. Hairpin-capped ends are the target of endonuclease activity by the Mre11-RAD50-Xrs2 and Sae2 complex. Because such endonuclease activities potentially disrupt BFB cycles, it is possible that some level of dysfunction in the endonuclease activity is required for sustaining BFB cycles in tumor cells.

We considered BFB cycles as a candidate mechanism because copy number heterogeneity is very common in the *ERBB2* gene amplification. Genomic approaches led Inaki *et al*. to propose tandem duplication of the *ERBB2* locus as an initiating mechanism^36^. In their study, some of the paired ends representing tandem duplications existed in tumor genomes at a very high copy number. Because breakpoints with a very high copy-number can derive from rearrangements that occurred early during amplification, the authors concluded that tandem duplication was likely an initiating event. Although this conclusion seems to be contradicted by our study, the two studies emphasized different features. Here, palindromic duplication was seen throughout the amplified *ERBB2* segments. Palindromic duplication in some case may have occurred early while others may have occurred late and existed only in a subpopulation of cells. Therefore, it is possible that, during the early stages of BFB cycles, some of the broken ends led to tandem duplication that became subsequently amplified. Alternatively, initial tandem duplication can be followed by BFB cycles. It is also important to point out that both studies have a small number of cases, with 9 cases in the study by Inaki *et al*. and 8 cases in our study. Studies with a large number of cases will be necessary to address these issues.

Treatment for HER2-positive breast tumors is among the most successful examples of personalized medicine, as the combination of target diagnosis and treatment strategy has been a routine for more than a decade in the clinic^28^,^29^,^30^. However, targeted therapy almost always provokes resistance^57^, and trastuzumab is no exception^32^,^33^. Furthermore, there are responders and non-responders to trastuzumab in a neoadjuvant setting^58^,^59^,^60^. In our experience, neoadjuvant trastuzumab results in a complete response at the time of surgery for one-third of HER2-positive tumors, whereas one-third of patients are left with significant tumor burdentumors (J.J., and A.G. unpublished). Gene amplification was originally discovered as a resistance mechanism to a drug resistance^61^ and plays a role in the resistance to other targeted therapies in primary tumors^4^,^5^,^62^. The amplification mechanism of *ERBB2* is a critical missing piece of information for the better understanding of the biology of HER2-positive breast tumors. A large-scale study that further promote our understanding of the *ERBB2* amplification mechanism is needed.

## Methods

### Ethics statement

Genomic studies using tumor DNA was approved under the Internal Institutional Review Board at the Cleveland Clinic (IRB07-136: EXEMPT: Chromosome Breakage and DNA Palindrome Formation). Specimens were obtained and methods were carried out under the auspices of IRB 7881 (Evaluation of Genetic and Molecular Markers in Patients with Breast Cancer). Informed consent was obtained from all participants to allow their cancer specimens to be used by researchers in an anonymized fashion. The consent form indicates that publication will take place without identifiers to protect the identity of any specific individual.

A study assessing copy number heterogeneity was approved under the Internal Institutional Review Board at the Cedars-Sinai Medical Center (Pro00037124: HER2 copy number status before and after neoadjuvant HER2-targeted therapy in HER2-positive breast tumors). FISH signals for both *ERBB2* and CEP17 were counted in 20 nuclei in each tumor slide. To evaluate the HER2 FISH, the invasive carcinoma was first circled on a corresponding H&E stained slide (microdissection). The microdissected area was scanned on the FISH slide. Cells from several representative areas were then counted for a total of 20 cells.

### Samples and DNA extraction

Breast tumor tissues were obtained as described previously^48^. Tissues were identified from the tissue archives in the Pathology Department at the Cleveland Clinic, specifically from consenting patients (IRB 7881). HER2 status of these tumors was determined by FISH. We first examined hematoxylin/eosin (HE)-stained sections and confirmed that at least 80% of cellularity was derived from tumor. Five 10-mm sections were subject to DNA extraction. To extract DNA, tissue sections were incubated in the lysis buffer (100 m*M* NaCl/10 mM Tris⋅HCl, pH 8.0/25 mM EDTA/0.5% SDS/proteinase K) for 24 hours at 37 °C. The solution was mixed with phenol/chloroform and DNA was precipitated from the aqueous phase by ethanol, as described previously^8^.

### Genome-wide analysis of palindrome formation (GAPF-seq) and array-comparative genomic hybridization

Raw data is available from the Database of Genotype and Phenotype under the study name “DNA inverted repeats as an at-risk motif of Palindromic Gene Amplification”.

Genomic DNA from primary breast tumors was processed for genome-wide analysis of palindrome formation (GAPF) as previously described with minor modifications^41^,^42^. Two micrograms (μg) of genomic DNA was used as starting material, of which 1 μg was digested with KpnI and 1 μg was digested with SbfI for 16 h (20 μl of reaction with 10 U of each enzyme). After the heat inactivation of the enzyme (65 °C for 20 min), digests were combined for denaturation (7 min in boiling water) and rapid renaturation in the presence of 100 mM NaCl and 50% formamide (90 μl of reaction mix consisting of 40 μl of DNA digests, 3 μl of 3 M NaCl, 45 μl of formamide and 2 μl of water). Following the digestion with nuclease S1 (Invitrogen) (120 μl of reaction consisting of 90 μl of DNA, 8 μl of 3 M NaCl, 12 μl of S1 nuclease buffer, 2 μl of 100 U/μl S1 nuclease and 8 μl of water), DNA was purified using the ChargeSwitch PCR Clean-up Kit.

Sequencing library construction and sequencing were done by the Genomics Facility at the University of Chicago. After fragmentation using the M220 focused ultrasonicator (Covaris), fragments between 100 and 250 bp were purified for NGS library construction using 5500 SOLiD™ Fragment Library Core Kit (Applied Biosystems). Each DNA sample was ligated to linkers with unique barcodes. Libraries were then pooled together and sequenced in a single flowcell run using a SOLiD 5500 × l sequencer. The barcodes were then used to trace the sequence data back to a specific sample. BAM files were generated using LifeTech LifeScope software. Read counts for each 5 kb bin was obtained using WIG files throughout the genome. We converted BAM files into WIG files using IGV tool. Read counts in tumor GAPF-seq data were normalized against the read counts in IMR90 (normal fibroblast) GAPF-seq data^39^. Genome-wide median (m) and median absolute deviation (s) of the normalized read counts (d) were determined. To obtain z-score for each 5 kb bin, the genome-wide mean was subtracted from the normalized read counts of a bin and divided by genome-wide median absolute deviation (d-ml/s)^63^.

Copy number analysis was done by the Gene Expression & Genotyping Core Facility of the Case Comprehensive Cancer Center (P30CA043703). One μg of genomic DNA was processed by the Genome-Wide Human SNP Nsp/Sty 5.0/6.0 Assay kit (Affymetrix) and hybridized to Affymetrix SNP6.0 arrays. Comparative data analysis and visualization were done using the Integrated Genome Viewer.

### Whole genome sequencing

Whole Genome sequencing for the breast tumor BT5 was done at the Genomics Facility of the University of Chicago. 5 μg of genomic DNA was processed for a library construction by TrueSeq DNA PCR-Free LT Sample Preparation Kit (Illumina). A genomic library was sequenced by HiSEQ 2500. An FASTQ file was converted into a SAM file using BWA. We only selected read pairs with the MAPQ score >10 for the analysis. For each 10 kb bin, we determined (1) the total number of mapped reads (/10 kb bin) (representing copy number) and (2) the number of reads whose paired reads were mapped on the same strand and less than 10 kb apart (representing fold-back inversions).

### Publically available WGS data

We obtained breast tumor WGS data from European Genome Phenome Archive (accession number EGAD00001001334)^43^. We estimated tumor/normal CN ratios using read depth analysis. We focused on 30 cases (PD6048, PD6404, PD6405, PD7202, PD7203, PD7204, PD7205, PD7304, PD7305, PD7306, PD7307, PD8998, PD9009, PD11344, PD11345, PD11347, PD11348, PD11349, PD11462, PD11464, PD11465, PD13163, PD13165, PD13167, PD13602, PD18407, PD18050, PD18188, PD18189), in which the CN ratio of the *ERBB2* locus was at least four times of the average CN ratio of the entire chromosome 17. We identified fold-back inversions by Genomon-SV using the information in chimeric reads as well as discordant paired-end reads, as described previously^44^.

## Additional Information

**How to cite this article**: Marotta, M. *et al*. Palindromic amplification of the *ERBB2* oncogene in primary HER2-positive breast tumors. *Sci. Rep.* **7**, 41921; doi: 10.1038/srep41921 (2017).

**Publisher's note:** Springer Nature remains neutral with regard to jurisdictional claims in published maps and institutional affiliations.

## Supplementary Material

Supplementary Figures

## Figures and Tables

**Figure 1:**
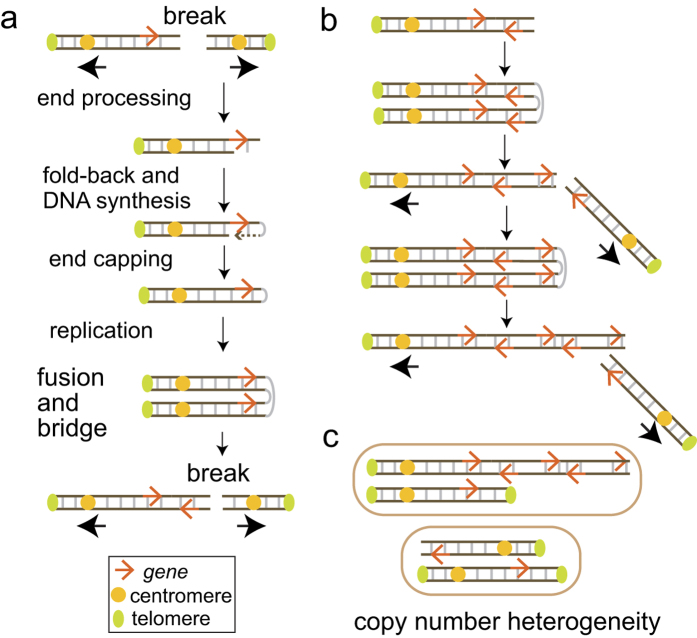
Palindromic duplication of a gene by Breakage-Fusion-Bridge cycles (model). (**a**) One cycle of Breakage-Fusion-Bridge. A dicentric chromosome breaks after each centromere is pulled to an opposite pole. End processing and fold-back DNA synthesis create a hairpin-capped chromosome. Replication of the hairpin-capped chromosome generates an isodicentric chromosome with the inverted duplication of *ERBB2* genes.(**b**) At (iso) dicentric breakage, each daughter cell receives an unequal amount of genetic material. (**c**) Copy number heterogeneity generated by BFB cycles. Each cell has an intact and a rearranged chromosome.
