# Supplementary material for: Palindromic amplification of the ERBB2 oncogene in primary HER2-positive breast tumors
Source: Sci Rep. 2017 Feb 17;7:41921. doi: 10.1038/srep41921 (PMC5314454; doi:10.1038/srep41921)
Supplement: Supplementary Figures [file srep41921-s1.pdf]

Supplemental Figures Table of Contents

Palindromic amplification of the *ERBB2* oncogene in human primary breast tumors

Michael Marotta[1,5], Taku Onodera[3,5], Jeffrey Johnson[4], Thomas Budd[2], Takaaki Watanabe[1,4], Xiaojiang Cui[4], Armando Giuliano[4], Atsushi Niida[3] and Hisashi Tanaka[1,4]

Figure 1 A common pattern of copy number transition of chromosome 17 in HER2-positive breast tumors

Figure 2 Procedure of GAPF-seq

Figure 3 Local genomic environment of the region surrounding *ERBB2* gene

Figure 4 Copy number alterations of chromosome 17 in HER2-positive breast cancer

Figure 5 Mechanisms for generating a hairpin-capped chromosome 17 (model)

Supplementary Figure 1 - Marotta *et al*,

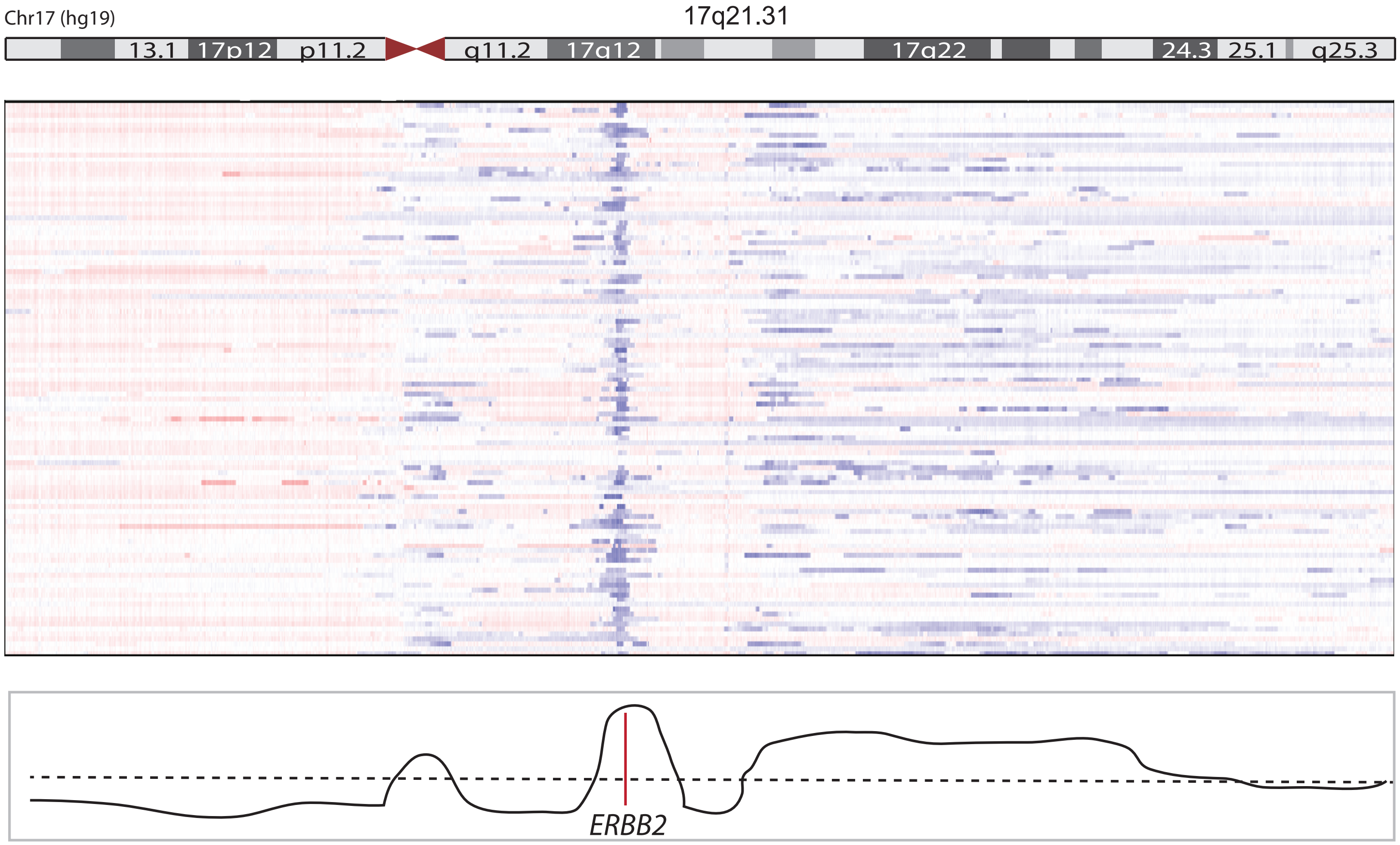

A common pattern of copy number transition of chromosome 17 in HER2-positive breast tumors
A heat map showing the copy number alterations in 117 HER2-positive breast tumors. Blue indicates genomic regions with copy-number gains and red indicates regions with copy-number loss.

Supplementary Figure 2 - Marotta *et al*,

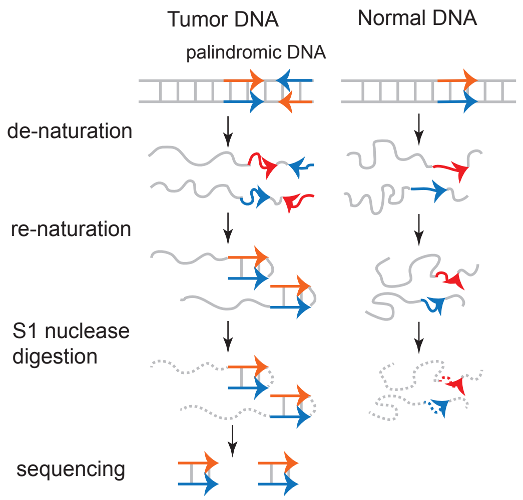

Procedure for enriching palindromic DNA from tumor genomic DNA. Palindromic DNA is shown by the pair of a blue and a red arrow that are complementary to each other. After denaturation and quick renaturation, palindromic DNA become double-stranded (dsDNA), while non-palindromic DNA remains single-stranded (ssDNA). Single-stranded nucleic acid specific nuclease S1 digests ssDNA, but not dsDNA. Thus the treatment by S1 results in the enrichment of DNA that derives from palindromic DNA.

Supplementary Figure 3 - Marotta *et al*,

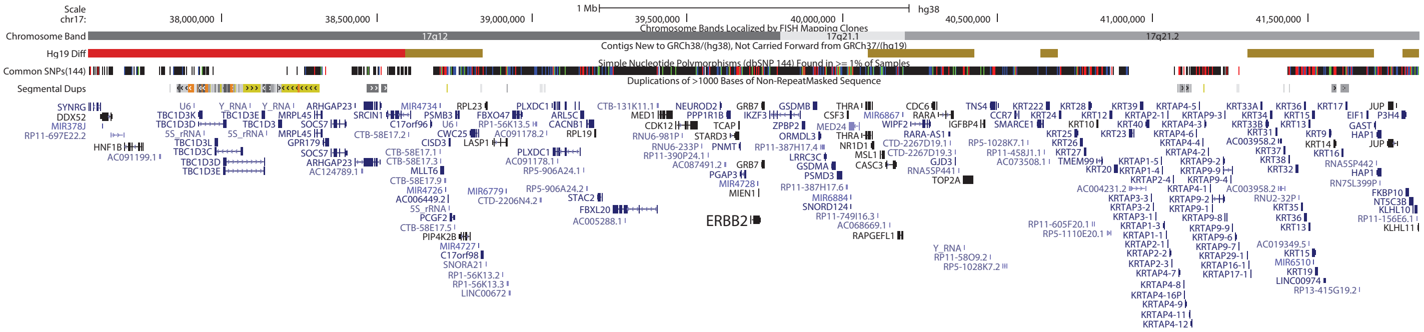

Local genomic environment of the surrounding *ERBB2* gene. Coordinates of chromosome 17, chromosome bands, contigs discordant from the previous genome assembly, dbSNPs, segmental duplications, and genes are shown (modified from a figure taken from UCSC genome browser). Note that the centromeric side is flanked by the large discordant contigs and a large block of segmental duplications, whereas the telomeric side is flanked by the mumber of KRTAP and KRT gene families. As a result, these regions lack definitive SNP markers.

Supplementary Figure 4 - Marotta *et al*,

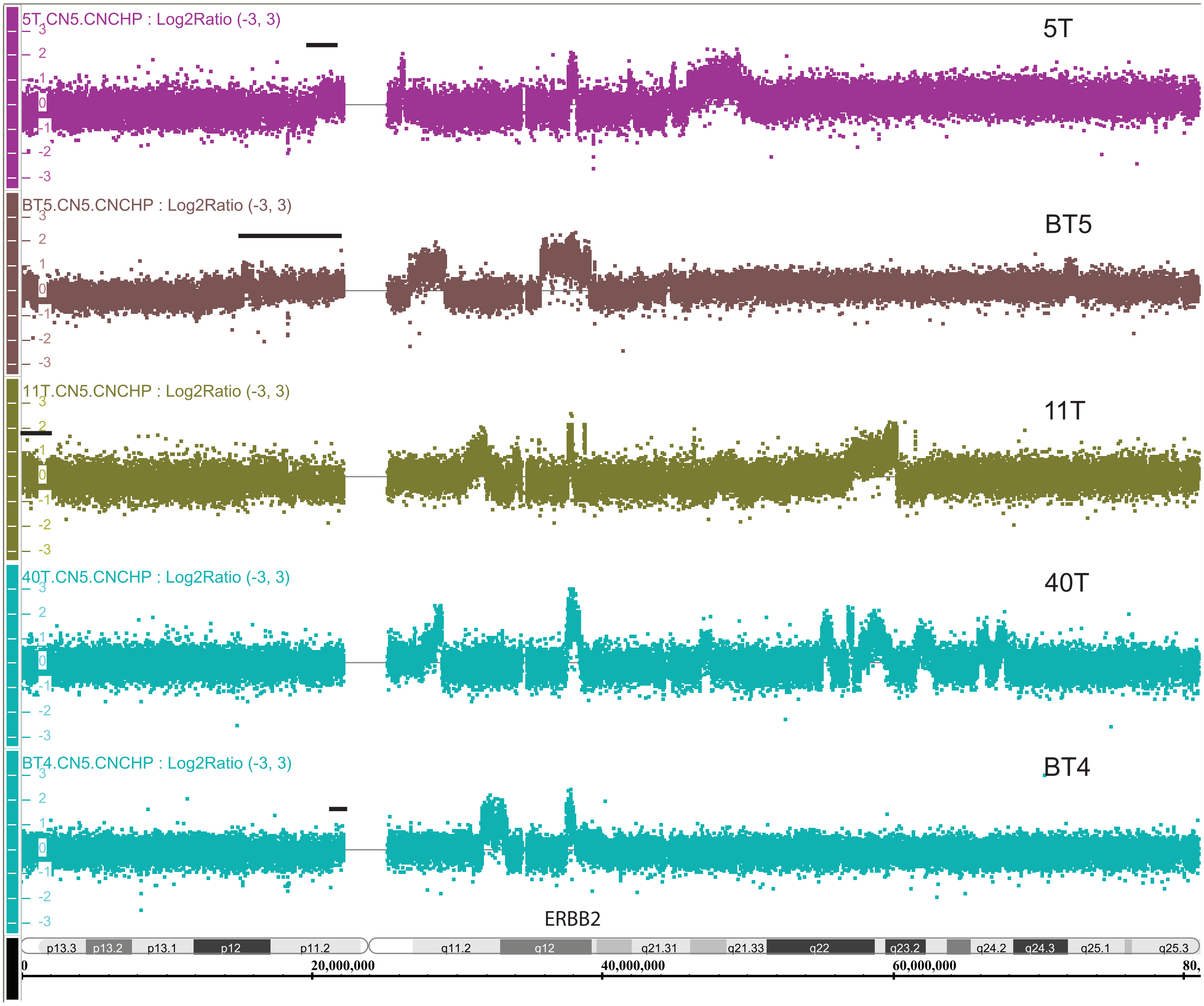

Copy number alterations of chromosome 17 in 5 HER2-positive breast tumors. The location of ERBB2 amplicon is shown on the chromatograph. The regions on the 17p that retain copy numbers are indicated by black lines.

Supplementary Figure 5 - Marotta *et al*,

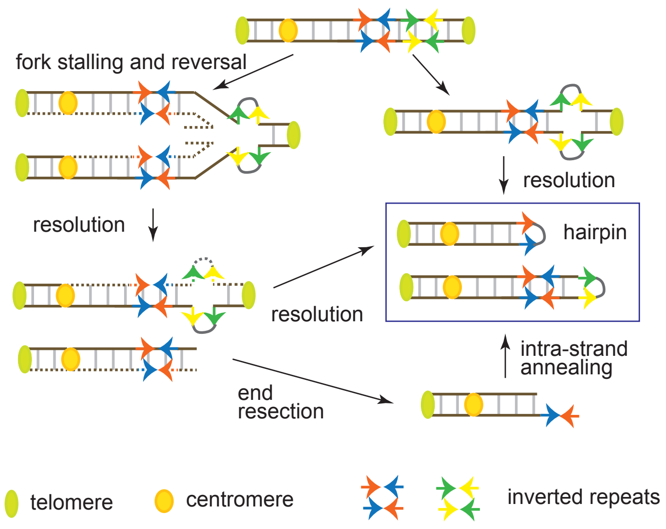

Inverted repeats-guided formation of a hairpin-capped chromosome (model). Replication forks stall at the inverted repeat (left). Fork reversal creates a four-way junction, the resolution of which results in a one-ended DSB. Processing of the end and intra-strand annealing lead to the formation of a hairpin-capped chromosome. Alternatively, a cruciform structure formed by the intra-strand annealing is resolved to form a hairpin-capped chromosome (right).
